# Analysis of deafness gene screening results in 15771 newborn cases in Anyang city of Henan

**DOI:** 10.3389/fped.2025.1645070

**Published:** 2025-10-16

**Authors:** Yanchao Mu, Meiqing Han, Yang Li, Hua Di, Zhengxin Li, Haiyan Li, Dawei Wang, Xue Li, Lei Zhang, Huiping An

**Affiliations:** ^1^ Prenatal Diagnosis Center, Anyang Key Laboratory of Prenatal Diagnosis, Anyang Maternal and Child Health Hospital, Anyang, China; ^2^ Laboratory Medicine Department, Anyang Maternal and Child Health Hospital, Anyang, China; ^3^ Neonatal ICU, Anyang Maternal and Child Health Hospital, Anyang, China; ^4^ Department of Health, Anyang Maternal and Child Health Hospital, Anyang, China; ^5^ Department of Otolaryngology, Anyang Maternal and Child Health Hospital, Anyang, China

**Keywords:** hereditary hearing loss, deafness gene, genetic screening, newborn, variant locus

## Abstract

**Objective:**

To analyze the prevalence and mutation spectrum of deafness-associated genes among newborns in Anyang City.

**Methods:**

Heel blood samples were collected from 15,771 newborns. Thirteen mutation sites across four genes associated with hereditary deafness (*GJB2, SLC26A4, GJB3, and 12S rRNA*) were detected using PCR combined with a flow-through hybridization technique.

**Results:**

A total of 605 newborns were identified as carriers of pathogenic variants, yielding an overall carrier rate of 3.836%. Specifically, 254 newborns carried *GJB2* gene variants (carrier rate: 1.611%), including one homozygous variant. Heterozygous variants in the *SLC26A4* gene were found in 257 newborns (carrier rate: 1.630%). Heterozygous GJB3 variants were detected in 49 newborns (carrier rate: 0.311%). Homoplasmic or heteroplasmic variants in the *12S rRNA* gene were present in 42 newborns (carrier rate: 0.266%). Additionally, ten newborns carried heterozygous variants in two different genes concurrently. Five *12S rRNA* variants found in this study were not documented in public databases. The frequency of deafness gene variants in descending order was *SLC26A4, GJB2, GJB3,* and *12S rRNA*. The most common pathogenic variants identified were *GJB2* c.235delC and c.299_300delAT, and *SLC26A4* c.919-2A > G and c.2168A > G, consistent with findings from other regions in China.

**Conclusion:**

Implementing newborn genetic screening for deafness in this region facilitates the early identification of individuals at risk for congenital, delayed-onset, and aminoglycoside-induced hearing loss, enabling timely intervention and follow-up.

## Introduction

Hereditary hearing loss (HHL), which accounts for over 60% of congenital deafness cases globally, is one of the most prevalent sensorineural disorders in neonates (1). It is a highly heterogeneous disorder, meaning it can be caused by mutations in many different genes and can present in a variety of ways. It can be congenital or have a delayed onset. The progression of HHL can be stable or progressive. The degree of hearing loss can range from mild to moderate, severe, or profound. HHL is most commonly bilateral and symmetrical. However, asymmetrical or even unilateral loss can occur. Type of hearing loss includes sensorineural hearing loss, conductive hearing loss, mixed hearing loss and association with other symptoms. Major types of HHL includes non-syndromic hearing loss and syndromic hearing loss. There are various known syndromes associated with hearing loss, such as Usher Syndrome, Pendred Syndrome, Waardenburg Syndrome, Jervell and Lange-Nielsen Syndrome, Alport Syndrome. Advances in molecular genetics and genomic technologies over the past three decades have revolutionized our understanding of HHL's genetic basis, enabling large-scale screening initiatives to mitigate its lifelong impact. This study traces the historical evolution of HHL research, elucidates the discovery of causative genes, discusses technological breakthroughs in genetic diagnostics, and underscores the imperative of population-based newborn screening, contextualizing our team's large-scale genetic screening initiative for neonatal deafness in Anyang City, Henan Province, China.

The recognition of hereditary deafness dates back to the 19th century when familial clustering of deaf individuals was first documented (2). Early studies relied on pedigree analysis, revealing autosomal recessive inheritance as the predominant pattern (∼80% of cases), with smaller proportions attributed to autosomal dominant, X-linked, and mitochondrial mutations (2, 3). However, the lack of molecular tools limited researchers to phenotypic classifications until the 1990s, when positional cloning identified *GJB2* (encoding connexin 26) as the first major HHL-associated gene (4). This breakthrough catalyzed the “Golden Age” of HHL gene discovery, with over 150 causative genes now implicated in syndromic and nonsyndromic hearing loss (5).

The post-genomic era accelerated the identification of HHL genes through genome-wide association studies (GWAS) and exome sequencing. Key discoveries include pathogenic variants in *SLC26A4*: Linked to Pendred syndrome and enlarged vestibular aqueduct (6), now recognized as the second most common cause of recessive HHL in East Asia. Mitochondrial mutations: The m.1555A > G variant in *MT-RNR1*, responsible for aminoglycoside-induced ototoxicity (7), highlights gene-environment interactions. *OTOF*-related auditory neuropathy: Biallelic *OTOF* mutations disrupt synaptic vesicle release in inner hair cells, accounting for 3%–10% of prelingual deafness (8). Population-specific mutations, such as GJB2 c.109G > A in Caucasians and c.235delC in East Asians, underscore the need for regionally tailored screening panels (4).

Diagnostic approaches evolved from laborious Sanger sequencing to next-generation sequencing (NGS). Targeted gene panels (50–200 genes) achieve diagnostic yields of 40%–60%. Whole-exome sequencing (WES) increases resolution to 65%–70%, particularly for atypical phenotypes. Third-generation sequencing resolves structural variations in challenging regions like *STRC*, a common pseudogene-interfered locus (9). Universal newborn hearing screening (UNHS), implemented in >90% of U.S. states and European Union nations, identifies auditory deficits but cannot distinguish genetic from acquired causes. Combining UNHS with genetic testing enhances etiological diagnosis, detecting actionable variants at a 0.1% rate (10). Population-based genetic screening is critical for preventing HHL in newborns. Neonatal identification of HHL enables timely interventions: Cochlear implantation before 12 months optimizes speech development, and cochlear implantation is a well tolerated and effective treatment for pediatric patients under the age of five years with single-sided deafness (11). Avoidance of ototoxic drugs in *MT-RNR1*, encoding the 12S ribosomal RNA (*12S rRNA*), carriers prevents iatrogenic deafness. The *12S rRNA* is a core structural and functional component of the mitochondrial ribosome (mitoribosome), essential for mitochondrial protein synthesis and, consequently, oxidative phosphorylation and cellular energy production. Highly conserved regions of this molecule are particularly vulnerable to pathogenic mutations that disrupt ribosomal assembly, fidelity, or interactions, leading to impaired mitochondrial translation and bioenergetic deficits within the metabolically demanding cells of the inner ear, ultimately triggering apoptotic pathways and irreversible HL. Carrier screening in high-prevalence populations reduces reproductive risk through genetic counseling. Modeling studies demonstrate that combined audio genetic screening saves 1,500–3,800 United States dollars per quality-adjusted life year (QALY) by averting delayed diagnoses in a low- to middle-income country (12). For China home to ten million annual births scaling up screening could prevent 20,000 cases of preventable deafness yearly.

Decades of research have transformed HHL from an enigmatic disorder into a preventable public health challenge. However, global disparities in genetic screening access persist, particularly in resource-limited regions. This newborn hereditary deafness genetic screening initiative pursues three interlocking scientific objectives: (1) determining regional prevalence of deafness-associated gene variants, informing evidence-based public health strategies for hereditary hearing loss prevention; (2) identifying novel pathogenic loci through population-level genomic analysis, thereby advancing molecular diagnostics and therapeutic target identification for next-generation deafness detection kits; (3) fostering interdisciplinary collaboration through establishing standardized data-sharing protocols that bridge epidemiological findings with clinical genomics across diverse geographical cohorts. These aims collectively address critical gaps in precision audiology while fostering technological innovation and global knowledge translation in congenital hearing disorder management.

## Materials and methods

### Subjects

This is a retrospective cohort study based on existing medical records. Using Anyang's Maternal and Child Health Information System, we identified and screened a patient cohort that met the inclusion criteria for our study based on deafness genetic screening, singleton birth, and household registration status. Our cohort does not fully represent the general population of Anyang. Instead, it represents a subpopulation of individuals in Anyang who had access to deafness genetic screening and were recorded in the city's Maternal and Child Health Information System between July 1, 2022, and October 31, 2024. Postpartum women with hearing loss were excluded (*n* = 15 771), 28.17 ± 4.61 (19–47) years old, 38.40 ± 1.52 (28–43) gestational weeks of delivery. To minimize potential confounding effects from genetic duplicates and multiple births, pregnancies involving twins or multiple births were excluded. This work described has been carried out in accordance with The Code of Ethics of the World Medical Association (Declaration of Helsinki) for experiments involving humans. Ethics Committee approval for this retrospective study (ID:20230225006).

### Experimental procedure

Blood Collection: Utilize the heel prick method for newborns to collect blood. Soak the filter paper circle on the blood collection card with the collected blood until saturated and allow it to dry for later use.

Sample Preparation: Punch out 6 pieces of 3 × 3 mm blood spots from the dried blood spot card and place them into a 1.5 ml centrifuge tube. Add 300 µl of sample release agent and 20 µl of Proteinase K to the tube. Mixed thoroughly and incubate at 65 °C for 20 min.

Nucleic Acid Extraction: DNA Extraction Kit and Hearing Loss Susceptibility GenoArray Diagnostic Kit (ID:20243400908) reagent were purchased from Hybribio Company (35/F, Enterprise Square Two, No. 3 Sheung Yuet Road, Kowloon Bay, Hong Kong). Power on the nucleic acid extraction instrument. Place the EP tube into the Fully Automated Nucleic Acid Extraction System (AutoPrep) (Hybribio Company) and select the program “DR-4801-KZ” for automated extraction.

Preparation of PCR Mixture: Allow the PCR mixture to thaw naturally at room temperature and mix thoroughly. Dispense 27.5 µl of the mixture per person and add 1 µl of DNA polymerase. Mix well and set aside for later use.

PCR Amplification: Take 28 µl of the prepared PCR mixture and add 2 µl of the extracted DNA solution into a PCR reaction tube. Mix well. Place the tube in the Automatic Medical PCR Analysis System (SLAN-96) (Hybribio Company) and set the following amplification program: 95 °C for 5 min; 40 cycles of 95 °C for 10 s, 55 °C for 30 s, and 72 °C for 1 min; final extension at 72 °C for 2 min; and a hold at 25 °C for 3 min. The amplified PCR product is used for hybridization.

Hybridization and Result Interpretation: Preheat the hybridization solution and elution buffer in a 45° Cywater bath. After preheating, place them into the Flow-through Hybridization (HybriMax) (Hybribio Company). Add the PCR amplification product according to the instrument's prompt.

Interpretation of results: Interpret the test results after the instrument completes its run. In case of no blue-purple spots appear for both the normal and mutant probes at a particular detection site, it may indicate the presence of a new mutation type at that site and first-generation sequencing should be performed on the vicinity of the site for further analysis.

### Bioinformatic analysis

The pathogenicity of identified genetic variants was assessed using an integrated approach leveraging three established bioinformatic databases: SIFT (Sorting Intolerant From Tolerant), PolyPhen-2 (Polymorphism Phenotyping v2), and ExAC (Exome Aggregation Consortium). These database provides distinct but complementary information regarding the potential functional impact or population frequency of missense variants. SIFT: SIFT predicts whether an amino acid substitution affects protein function based on sequence homology and the conservation of residues across related protein sequences. The algorithm generates a normalized probability score ranging from 0.0 (deleterious) to 1.0 (tolerated). Variants with SIFT scores ≤0.05 were classified as “Deleterious” (intolerant to substitution), indicating a high probability of impacting protein function and potential pathogenicity. Variants with scores >0.05 were classified as “Tolerated”. PolyPhen-2: PolyPhen-2 employs a combination of sequence-based features, phylogenetic conservation, and structural parameters within a supervised machine-learning framework to predict the possible impact of an amino acid substitution. Predictions are categorized qualitatively as: Probably Damaging, Possibly Damaging or Benign. ExAC: The ExAC serves as a critical filter for identifying rare variants unlikely to be benign polymorphisms. We primarily utilized the ExAC non-Finnish European (NFE) subpopulation frequency and the overall global minor allele frequency (MAF). Variants with a global MAF > 0.1% (MAF > 0.001) in ExAC were generally considered unlikely to be highly penetrant pathogenic mutations for severe disorders and were thus deprioritized unless compelling functional or segregation evidence suggested otherwise. Conversely, variants with MAF < 0.1% (especially absent or extremely rare, MAF ≪ 0.001) were considered more plausible candidates for pathogenicity. Referring to the classification standards and guidelines of genetic variations of the American College of Medical Genetics and Genomics (ACMG), the pathogenicity of the test data was interpreted.

## Results

### Baseline results

The baseline characteristics of pregnant women in the study cohort are summarized in Table 1 top. Demographic and clinical parameters, including age at delivery (28.17 ± 4.61, 19–47 years), gestational week at delivery (38.40 ± 1.52, 28–43 weeks), weight (59.67 ± 8.31,45–95 kg), height (161.65 ± 4.82, 145–180 cm), age of menarche (14.21 ± 2.62, 10–24 years), menstrual cycle (26.15 ± 12.18, 18–79 days), complications of pregnancy (509, 3.23%), and lifestyle factors such as cigarette use (126 (126, 0.80%), alcohol consumption (1,055, 6.69%). The baseline characteristics of newborns in the study cohort are presented in Table 1 bottom. Clinical parameters included neonatal Apgar score (9.27 ± 0.98, 0–10), birth weight, (3.27 ± 0.49, 0.58–5.80 kg), birth length, (50.27 ± 2.32, 25.0–66.0 cm), gestational age (38.40 ± 1.52, 28–43week), and so on.

**Table 1:** Baseline characteristics of pregnant women and newborns.

Subject	Column	Data
pregnant women (*n* = 15 771)	Age of delivery, year	28.17 ± 4.61 (19–47)
	Gestational week of delivery, week	38.40 ± 1.52 (28–43)
	Weight, kg	59.67 ± 8.31 (45–95)
	Height, cm	161.65 ± 4.82 (145–180)
	Age of menarche, year	14.21 ± 2.62 (10–24)
	Menstrual cycle, days	26.15 ± 12.18 (18–79)
	Complications of pregnancy, *n* (%)	509 (3.23%)
	Cigarette, *n* (%)	126 (0.80%)
	Alcohol, *n* (%)	880 (5.58%)
	Medicine, *n* (%)	1,055 (6.69%)
Newborns (*n* = 15 771)	Male	8,011 (50.80%)
	Female	7,760 (49.20%)
	Neonatal Apgar score	9.27 ± 0.98 (0–10)
	Birth weight, kg	3.27 ± 0.49 (0.58–5.80)
	Birth length, cm	50.27 ± 2.32 (25.0–66.0)
	Gestational age, week	38.40 ± 1.52 (28–43)
	Caesarean birth	8,737 (55.40%)
	Vaginal birth	7,034 (44.62%)

### Screening results

In this study, a total of 605 newborns were identified as carriers of pathogenic variants, resulting in an overall carrier rate of 3.836%. 254 newborns carried GJB2 gene variants (carrier rate: 1.611%), including 1 homozygous variant. Heterozygous variants in the *SLC26A4* gene were found in 257 newborns (carrier rate: 1.630%). Heterozygous *GJB3* variants were detected in 49 newborns (carrier rate: 0.311%). Homoplasmic or heteroplasmic variants in the *12S rRNA* gene were present in 42 newborns (carrier rate: 0.266%). Additionally, 10 newborns carried heterozygous variants in two different genes concurrently. Five *12S rRNA* variants found in this study were not documented in public databases: m.7247C > T, m.7433C > T, m.7439A > G, m.7441C > T, and m.7447A > G. The frequency of deafness gene variants in descending order was *SLC26A4, GJB2, GJB3,* and *12S rRNA*. The most common pathogenic variants identified were *GJB2* c.235delC and c.299_300delAT, and *SLC26A4* c.919-2A > G and c.2168A > G in this study. See Table 2 for details.

**Table 2:** Deafness gene screening results in 15 771 newborn cases.

Gene	Transcript	Genotype	SIFT	Polyphen2	ExAC	Zygosity	ACMG	Case [*n*, %, (95% CI%)]
*GJB2*	NM_004004	c.176_191del16	Damaging	Probably Damaging	1.6 × 10^−05^	Heterozygous	Pathogenic	13, 0.082, (0.048, 0.141)
		c.235delC	Damaging	Probably Damaging	4.0 × 10^−4^	Homozygous	Pathogenic	1, 0.006, (0.001, 0.036)
		c.235delC	Damaging	Probably Damaging	4.0 × 10^−4^	Heterozygous	Pathogenic	197, 1.249, (1.083, 1.432)
		c.235delC, c.109G > A	Damaging	Probably Damaging	–	Heterozygous, Heterozygous	Pathogenic	1, 0.006, (0.001, 0.036)
		c.299_300delAT	Damaging	Probably Damaging	4.1 × 10^−05^	Heterozygous	Pathogenic	34, 0.216, (0.150, 0.302)
*GJB2, 12S rRNA*	NM_004004 NC_012920	c.235delC, m.7440T > G	Damaging	Probably Damaging	–	Heterozygous, Homogeneous	Pathogenic	1, 0.006, (0.001, 0.036)
		c.235delC, m.1555A > G	Damaging	Probably Damaging	–	Heterozygous, Homogeneous	Pathogenic	1, 0.006, (0.001, 0.036)
*GJB2, SLC26A4*	NM_004004 NM_000441	c.235delC, c.919-2A > G	Damaging	Probably Damaging	–	Heterozygous, Heterozygous	Pathogenic	3, 0.019, (0.006, 0.056)
		c.299_300delAT, c.919-2A > G	Damaging	Probably Damaging	–	Heterozygous, Heterozygous	Pathogenic	2, 0.013, (0.003, 0.046)
*GJB2, GJB3*	NM_004004 NM_024009	c.235delC, c.538C > T	Damaging	Probably Damaging	–	Heterozygous, Heterozygous	Pathogenic	1, 0.006, (0.001, 0.036)
*SLC26A4*	NM_000441	c.1229C > T	Damaging	Probably Damaging	2.0 × 10^−4^	Heterozygous	Pathogenic	12, 0.076, (0.040, 0.133)
		c.2168A > G	Damaging	Probably Damaging	1.0 × 10^−4^	Heterozygous	Pathogenic	27, 0.171, (0.114, 0.249)
		c.919-2A > G	Damaging	Probably Damaging	3.0 × 10^−4^	Heterozygous	Pathogenic	212, 1.344, (1.173, 1.533)
*SLC26A4, 12S rRNA*	NM_000441 NC_012920	c.2168A > G m.12196C > T	Damaging	Probably Damaging	–	Heterozygous, Homogeneous	Pathogenic	1, 0.006, (0.001, 0.036)
*GJB3*	NM_024009	c.538C > T	Damaging	Probably Damaging	8.238 × 10^−05^	Heterozygous	Pathogenic	47, 0.298, (0.220, 0.397)
*GJB3* *SLC26A4*	NM_024009 NM_000441	c.538C > T c.919-2A > G	Damaging	Probably Damaging	–	Heterozygous, Heterozygous	Pathogenic	1, 0.006, (0.001, 0.036)
*12S rRNA*	NC_012920	m.1494C > T	Damaging	Probably Damaging	–	Homogeneous	Pathogenic	2, 0.013, (0.003, 0.046)
		m.1555A > G	Damaging	Probably Damaging	–	Homogeneous	Pathogenic	5, 0.095, (0.014, 0.074)
		m.1555A > G	Damaging	Probably Damaging	–	Heterogeneous	Pathogenic	2, 0.013, (0.003, 0.046)
		m.7247C > T*	Tolerated	Possibly Damaging	–	Homogeneous	VUS	1, 0.006, (0.001, 0.036)
		m.7256C > T	Tolerated	Benign	–	Homogeneous	Benign	1, 0.006, (0.001, 0.036)
		m.7433C > T*	Tolerated	Possibly Damaging	–	Homogeneous	VUS	3, 0.019, (0.006, 0.056)
		m.7439A > G*	Tolerated	Possibly Damaging	–	Homogeneous	VUS	2, 0.013, (0.003, 0.046)
		m.7440T > C	Tolerated	Benign	–	Homogeneous	VUS	1, 0.006, (0.001, 0.036)
		m.7441C > T*	Tolerated	Possibly Damaging	–	Homogeneous	VUS	2, 0.013, (0.003, 0.046)
		m.7443A > G	Tolerated	Possibly Damaging	–	Homogeneous	Likely Pathogenic	1, 0.006, (0.001, 0.036)
		m.7444G > A	Tolerated	Benign	–	Homogeneous	Benign	1, 0.006, (0.001, 0.036)
		m.7445A > C	Tolerated	Possibly Damaging	–	Homogeneous	Pathogenic	1, 0.006, (0.001, 0.036)
		m.7447A > G*	Tolerated	Possibly Damaging	–	Homogeneous	VUS	1, 0.006, (0.001, 0.036)
		m.12192G > A	Tolerated	Benign	–	Homogeneous	VUS	9, 0.057, (0.030, 0.108)
		m.12193A > G	Tolerated	Benign	–	Homogeneous	Likely Benign	2, 0.013, (0.003, 0.046)
		m.12196C > T	Tolerated	Benign	–	Homogeneous	Likely Benign	3, 0.019, (0.006, 0.056)
		m.12206C > A	Tolerated	Benign	–	Homogeneous	VUS	2, 0.013, (0.003, 0.046)
		m.12338T > C	Tolerated	Benign	–	Homogeneous	VUS	1, 0.006, (0.001, 0.036)
		m.12361A > G	Tolerated	Benign	–	Homogeneous	VUS	1, 0.006, (0.001, 0.036)
Total								605, 3.836

* The asterisk indicates novel gene variants not yet included in the database. VUS, variant of unknown significance; CI, confidence intervals.

## Discussion

Neonatal screening for genetic variants associated with hearing loss represents a critical preventive strategy. Early identification facilitates timely interventions to mitigate speech development impairment secondary to deafness (preventing speech delay associated with deafness), reduces the incidence of delayed-onset hearing loss, provides essential medication guidance for at-risk individuals to prevent aminoglycoside-induced ototoxicity, and offers crucial information for future family planning (13). In our cohort of 15,771 newborns, we identified 605 carriers of pathogenic variants across four major deafness-associated genes, yielding a carrier rate of 3.836%. The primary genes investigated included *GJB2, SLC26A4, GJB3,* and *12S rRNA*, which are known to be significant contributors to genetic deafness in the Chinese population. In the Chinese population, the carrier rates of common hereditary hearing loss variants are as follows:GJB2 variants: c.235delC: ∼1.80%, c.299_300delAT: ∼0.50%, c.176del16: ∼0.12%, c.35delG: ∼0.01%, c.109G > A: ∼6.93%; SLC26A4 variants: c.919-2A > G: ∼1.34%, c.2168A > G: ∼0.27%, c.1229C > T: ∼0.08%; 12S rRNA variants: m.1555A > G: ∼0.21%, m.1494C > T: ∼0.02%, m.7445A > C: ∼0.02%, m.12201T > C: ∼0.01%; c.538C > T: ∼0.30% (14).

Consistent with its role as the leading common genetic cause of non-syndromic hearing loss (NSHL) in China, *GJB2* variants (autosomal recessive inheritance, population carrier rate 2%–3%) were the most frequently identified in our study. The protein product, Connexin 26 (Cx26), forms gap junction channels critical for potassium ion K^+^ recycling within the cochlea (15). Pathogenic variants, most notably c.235delC, disrupt channel function, leading to K^+^ accumulation, hair cell toxicity, and consequent hearing impairment. While homozygous or compound heterozygous variants typically cause congenital severe-to-profound deafness, phenotypic variability—including delayed onset—has been observed (16). Our screening identified one homozygous, one compound heterozygous, eight complex heterozygous, and 244 heterozygous carriers, with c.235delC, c.299_300delAT, and c.176_191del16 being the predominant variants. Neonates with biallelic pathogenic GJB2 variants require immediate audiological assessment and early intervention. Cochlear implantation, for instance, demonstrates excellent outcomes in this group and is crucial for preventing speech delays. Heterozygous carriers warrant genetic counseling, familial segregation analysis, and periodic hearing monitoring. *SLC26A4*, the second most prevalent deafness gene in our cohort, encodes pendrin, a chloride/iodide transporter essential for endolymphatic fluid homeostasis in the inner ear (17). Pathogenic variants, predominantly c.919-2A > G, cause protein misfolding and mislocalization, disrupting endolymph absorption. This leads to enlarged vestibular aqueduct (EVA) and Pendred syndrome (hearing loss with thyroid goiter) or non-syndromic EVA (18). We identified 250 heterozygous carriers, seven compound heterozygous carriers, but no homozygous carriers. Newborns with biallelic *SLC26A4* variants require urgent audiological and genetic confirmation and strict precautions to prevent head trauma, barotrauma, and vigorous activities known to trigger sudden hearing loss. Even heterozygous carriers should receive counseling on these risk-avoidance strategies, as they may have an increased susceptibility or be carriers for familial variants. *GJB3* variants (autosomal dominant or recessive) were less common, consistent with their lower population frequency. We identified 49 carriers of variants such as c.538C > T (p.Arg180Ter). *GJB3* encodes Cx31, expressed in the cochlea and auditory nerve (19). Variants disrupt gap junction function and ionic balance, typically manifesting as progressive high-frequency hearing loss in young adulthood. Regular audiological monitoring is advised for carriers. Mitochondrial *12S rRNA* variants (maternally inherited), particularly m.1555A > G and m.1494C > T, confer hypersensitivity to aminoglycoside antibiotics (20). We detected 44 carriers (carrier rate 0.28%), aligning with national data (∼0.29%). These variants alter the rRNA structure, creating aminoglycoside binding sites that induce mitochondrial dysfunction and irreversible hair cell death. Carriers may exhibit normal hearing or progressive loss; strict avoidance of aminoglycosides is paramount. Genetic counseling should emphasize this risk to the maternal lineage. Notably, we detected five *12S rRNA* variants not documented in public databases, suggesting potential novel variants. Research on these newly identified variants will facilitate pathogenicity assessment and elucidate novel mechanisms underlying hereditary hearing loss, serving as a critical foundation for diagnostic kit development. A significant finding was the identification of 11 neonates carrying pathogenic variants in two different deafness genes (e.g., *GJB2* and *SLC26A4*, *GJB2* and *12S rRNA*, etc.). This dual carriage complicates phenotype prediction and necessitates comprehensive genetic diagnosis, integrated audiological surveillance, long-term follow-up, and specialized genetic counseling to address potential synergistic effects or complex inheritance patterns. Proactive management offers these individuals the best chance for optimal hearing and speech outcomes and informs their future reproductive choices. A significant proportion of the variants reported in this study were classified as VUS. According to current ACMG guidelines, VUS should not be considered pathogenic variants and cannot serve as a basis for diagnosis. These findings are primarily documented for future research reference. For individuals carrying a VUS, clinical management should be based on phenotype and family history, rather than on the genetic test result itself. Confirmatory testing for the specific VUS in the proband's family members is recommended to obtain segregation data, which may aid in future variant reclassification. As more population data and functional evidence accumulate, the classification of some VUS is likely to change.

Recent advancements in genetic testing technologies, particularly next-generation sequencing (NGS), have significantly enhanced the accuracy and efficiency of genetic deafness diagnosis. NGS enables simultaneous detection of multiple gene variants, providing a comprehensive genetic profile and identifying rare and novel mutations that traditional methods may miss (21). However, the use of genetic testing in neonatal screening raises ethical and legal considerations, including privacy protection, genetic discrimination, and informed consent. Ensuring strict confidentiality of genetic information and fully informing patients and families about testing implications are essential. While genetic testing offers substantial benefits for early diagnosis and management of genetic deafness, cost remains a barrier to widespread adoption. Cost-effectiveness analyses suggest that the long-term benefits of early intervention and prevention of hearing loss outweigh the initial costs of genetic testing. Strategies to reduce costs, such as economies of scale and technological advancements, are needed to make genetic testing more accessible (22). The global shift toward personalized medicine and genetic testing is driving the development of more accurate and efficient diagnostic tools. International collaborations and data sharing are vital for advancing our understanding of genetic deafness and improving diagnostic capabilities. Standardizing testing protocols and sharing genetic data can enhance accuracy and reliability. Raising public awareness about genetic deafness and the importance of neonatal screening is crucial. Educational campaigns targeting healthcare providers, parents, and the general public can increase understanding and acceptance of genetic testing (23). Community involvement and support groups can also play a significant role in providing information and support to affected families. Future research should focus on expanding the genetic panel to include less common but clinically significant deafness genes. The development of more affordable and accessible genetic testing platforms is also needed to ensure widespread adoption. Longitudinal studies are required to assess long-term outcomes of early intervention and the impact of genetic testing on the quality of life.

Neonatal genetic deafness gene screening is a valuable tool for the early identification and management of hearing loss. This study highlights the prevalence of common deafness genes in the Chinese population and underscores the importance of comprehensive genetic testing. Advances in genetic testing technologies, coupled with increased public awareness and international collaborations, hold promise for improving the diagnosis and management of genetic deafness. Our study successfully demonstrates the feasibility and clinical utility of large-scale neonatal deafness gene screening. However, the panel was limited to these four major genes. These genes account for a substantial proportion of hereditary hearing loss in the Chinese population, our approach undoubtedly underestimates the total genetic burden. Variants in other important genes, such as *OTOF, CDH23, TMC1* and *MYO15A*, which are associated with both syndromic and non-syndromic hearing loss, were not detected. Future studies employing expanded gene panels or whole-exome sequencing are warranted to obtain a more comprehensive genetic landscape in this cohort. Expanding screening via next-generation sequencing (NGS) to include other established (e.g., *MYO15A, OTOF, CDH23, TMC1*) and emerging deafness genes would significantly enhance diagnostic yield and capture a broader spectrum of genetic causes. In this study, audiological follow-up was conducted, and no significant differences in hearing outcomes were observed among the carrier population. We consider that extended follow-up may be necessary to potentially identify significant differences. Furthermore, long-term longitudinal studies are essential to fully evaluate the impact of early genetic diagnosis and intervention on developmental outcomes, quality of life, and cost-effectiveness. The results of this study are more suitable for describing the frequency characteristics of deafness gene carrier in a single population in Anyang area. Caution is needed when extending it to the absolute general population. Despite this limitation, our findings robustly support the integration of genetic screening with universal newborn hearing screening (UNHS) as a powerful strategy for the early detection, intervention, and prevention of hereditary hearing loss and its profound sequelae.

## Data Availability

The original contributions presented in the study are included in the article/Supplementary Material, further inquiries can be directed to the corresponding author.
